# Development and validation of a clinical prediction model for detecting coronary heart disease in middle-aged and elderly people: a diagnostic study

**DOI:** 10.1186/s40001-023-01233-0

**Published:** 2023-09-25

**Authors:** Shiyi Tao, Lintong Yu, Deshuang Yang, Ruiqi Yao, Lanxin Zhang, Li Huang, Mingjing Shao

**Affiliations:** 1 Graduate School, Beijing University of Chinese Medicine, Beijing, China; 2 Department of Integrative Cardiology, China-Japan Friendship Hospital, Beijing, China; 3 Department of Internal Medicine, Shenzhen Nanshan Chinese Medicine Hospital, Guangdong, China; 4 Department of Oncology, Guang’anmenHospital, China Academy of Chinese Medical Sciences, Beijing, China

**Keywords:** Coronary heart disease, Clinical prediction model, Nomogram, Risk factor, Diagnostic study

## Abstract

**Objective:**

To develop and validate a multivariate prediction model to estimate the risk of coronary heart disease (CHD) in middle-aged and elderly people and to provide a feasible method for early screening and diagnosis in middle-aged and elderly CHD patients.

**Methods:**

This study was a single-center, retrospective, case–control study. Admission data of 932 consecutive patients with suspected CHD were retrospectively assessed from September 1, 2020 to December 31, 2021 in the Department of Integrative Cardiology at China-Japan Friendship Hospital. A total of 839 eligible patients were included in this study, and 588 patients were assigned to the derivation set and 251 as the validation set at a 7:3 ratio. Clinical characteristics of included patients were compared between derivation set and validation set by univariate analysis. The least absolute shrinkage and selection operator (Lasso) regression analysis method was performed to avoid collinearity and identify key potential predictors. Multivariate logistic regression analysis was used to construct a clinical prediction model with identified predictors for clinical practice. Bootstrap validation was used to test performance and eventually we obtained the actual model. And the Hosmer–Lemeshow test was carried out to evaluate the goodness-fit of the constructed model. The area under curve (AUC) of receiver operating characteristic (ROC), calibration curve, decision curve analysis (DCA), and clinical impact curve (CIC) were plotted and utilized with validation set to comprehensively evaluate the predictive accuracy and clinical value of the model.

**Results:**

A total of eight indicators were identified as risk factors for the development of CHD in middle-aged and elderly people by univariate analysis. Of these candidate predictors, four key parameters were defined to be significantly related to CHD by Lasso regression analysis, including age (OR 1.034, 95% CI 1.002 ~ 1.067, *P* = 0.040), hemoglobin A1c (OR 1.380, 95% CI 1.078 ~ 1.768, *P* = 0.011), ankle-brachial index (OR 0.078, 95% CI 0.012 ~ 0.522, P = 0.009), and brachial artery flow-mediated vasodilatation (OR 0.848, 95% CI 0.726 ~ 0.990, *P* = 0.037). The Hosmer–Lemeshow test showed a good calibration performance of the clinical prediction model (derivation set, *χ*^*2*^ = 7.865, *P* = 0.447; validation set, *χ*^*2*^ = 11.132, *P* = 0.194). The ROCs of the nomogram in the derivation set and validation set were 0.722 and 0.783, respectively, suggesting excellent predictive power and suitable performance. The clinical prediction model presented a greater net benefit and clinical impact based on DCA and CIC analysis.

**Conclusion:**

Overall, the development and validation of the multivariate model combined the laboratory and clinical parameters of patients with CHD, which could be beneficial to the individualized prediction of middle-aged and elderly people, and helped to facilitate clinical assessments and decisions during treatment and management of CHD.

**Supplementary Information:**

The online version contains supplementary material available at 10.1186/s40001-023-01233-0.

## Introduction

Coronary heart disease (CHD) is a type of ischemic heart diseases characterized by atherosclerotic plaque accumulation in the coronary arteries [1] As one of the leading causes of hospitalization and death [2] CHD affects over 110 million individuals worldwide [3] and thus gives rise to a heavy burden on health expenditures [4] Advancing age is a major risk factor for cardiovascular events,[5, 6] previous evidence showed a higher incidence of CHD in males over 40 years old and the prevalence can be as high as 27.8% in patients over 60 years old [7] Currently, coronary angiography (CAG) is the reference standard for diagnosing CHD. Whereas, the more complications and worse physical conditions in elder people, including renal insufficiency, coagulation abnormalities, or intolerance to CAG [1] all of which require special attention, highlight the importance of alternative diagnostic methods that are more appropriate. Therefore, we try to establish a cardiovascular disease prediction model for this special group to provide a feasible method for early screening and diagnosis of high-risk patients.

Clinical prediction models are mathematical equations that relate multiple predictors to evaluate he probability of an outcome [8, 9] which can be used to gain insights into causality of the outcome of interest and have been recognized as reliable tools for quantifying risk in diagnostic and prognostic analyses [10, 11] Besides, nomogram is a prediction tool with the advantages of being graph-based and easy-to-understand, which can predict individualized specific risks for each patient in complex clinical settings [12, 13] And they could be valuable decision support tools to assist clinicians in the complicated choices they make regarding patient management.

We therefore developed and validated a diagnostic model combining the clinical and laboratory parameters of CHD in middle-aged and elderly people based on the clinical data of 839 eligible patients, to determine whether these factors could be incorporated into the model to provide a potential auxiliary solution for patient identification of CHD.

## Materials and methods

### Sampling design

This study was a single-center, retrospective, case–control study. Admission data of consecutive patients with suspected CHD between September 1, 2020 and December 31, 2021 were retrospectively assessed in the Department of Integrative Cardiology at China-Japan Friendship Hospital, which were obtained from the electronic medical records system and analyzed anonymously. All eligible patients were classified as the derivation set and validation set at a 7:3 ratio, respectively. Based on the results of CAG, patients with at least one coronary artery stenosis ≥ 50% were designated in the CHD group, and the rest were assigned to the non-CHD group in the two sets.

Inclusion criteria were as follows: (1) all patients with CHD met the diagnostic criteria from the guideline; [1, 14] (2) the age of the patient ≥ 45 years old; (3) every patient voluntarily signed informed consent for admission. Patients were excluded if they met one or more of the following criteria: (1) patients who suffered from severe cerebrovascular diseases, severe liver and kidney dysfunction, acute infection, malignant tumor, severe diseases of the endocrine and hematopoietic systems, mental diseases, pregnancy status, and patients in lactation, etc; (2) patients with contraindications to CAG or who cannot cooperate with the arterial vascular examination; (3) those without complete clinical data. This study was performed in accordance with the TRIPOD statement [15] and the Declaration of Helsinki of 1975, as revised in 2013.

### Sample size

The sample size for logistic regression is calculated by the following suggested Eq. (1).1

In Eq. (1), parameter  represents the number of independent variables and parameter  represents the smallest proportion of positive or negative cases in the population. As far as we know, the incidence of CHD in middle-aged and elderly people is about 27.8% [7] and the incidence is estimated to be 30% for the sample size calculation. Firstly, 100 samples were preliminarily included and analyzed and 9 variables were found to be significantly related to CHD after univariate analysis. Therefore, the minimum number of eligible patients required of the derivation set is: N = 10*9/0.3 = 300.

### Diagnostic criteria and of CHD

According to the “2019 ESC Guidelines for the diagnosis and management of chronic coronary syndromes” [1] and “Nomenclature and criteria for diagnosis of ischemic heart disease. Report of the Joint International Society and Federation of Cardiology/World Health Organization task force on standardization of clinical nomenclature” [14] CHD was diagnosed when CAG showed ≥ 50% stenosis in at least one coronary artery. CAG was conducted by a team of professional cardiologists in this study.

### Data collection

#### Basic information

The basic clinical information of all included patients was recorded, including demographic information (sex, age, height, weight, smoking history, etc.), clinical characteristics (coronary lesions, main symptoms, comorbidities, family histories, prior medication use, etc.). Peripheral venous blood samples were drawn in the fasting state in the morning of the 2nd day after admission and processed within 2 h. Serum indicators, including alanine aminotransferase (ALT), aspartate aminotransferase (AST), hypersensitive C-reactive protein (Hs-CRP), Homocysteine (HCY), total cholesterol (TC), triglyceride (TG), low-density lipoprotein cholesterol (LDL-C), high-density lipoprotein cholesterol (HDL-C), serum creatinine (Scr), N-terminal pro-B-type natriuretic peptide (NT-proBNP), and hemoglobin A1c (HbA1c) were tested in the Department of Clinical Laboratory of China-Japan Friendship Hospital.

#### Transthoracic echocardiography

Echocardiography was then performed and echocardiographic indexes consisting of left atrium diameter (LAD), left ventricular ejection fraction (LVEF), left ventricular end-diastolic diameter (LVDd), left ventricular posterior wall thickness (PWT), and interventricular septal thickness (IVST) were analyzed and recorded by two independent echocardiographers. Left ventricular mass (LVM) was determined using the anatomically validated Devereux equation [16] and normalized by body surface area (BSA), according to the formulas as follows: (1) LVM(g) = 0.8 × {1.04 × [(LVDd + PWT + IVST)^3^-LVDd^3^]} + 0.6; (2) BSA(m^2^) = 0.0061 ×  height (cm) + 0.0128 × weight (kg)-0.1529; (3) LVMI = LVM/BSA.

#### Measurements of baPWV and ABI

The limb arterial elasticity was examined before CAG in the morning, and patients abstained from alcohol-, nicotine-, caffeine-containing products, and vasodilator drugs for at least 8 h. All eligible patients were placed in a supine position after having 5 or more minutes of rest, with the limbs fully exposed. On both the left and right sides, the pressure cuff was attached to the brachial artery and the posterior tibial arteries, and then limb artery pulse waveform and blood pressure were measured and recorded automatically by a BP-203 RPEII arteriosclerosis detector (OMRON, Japan). Finally, the values of ankle-brachial index (ABI) and brachial-ankle pulse wave velocity (baPWV) were calculated. Meanwhile, heart rate, blood pressure, pulse volume waveform, and electrocardiogram were noted simultaneously during the test.

#### Measurement of FMD

All patients were positioned in a supine position after having 5 or more minutes of rest, with the right upper limb fully exposed, and monitored by limb lead electrocardiogram continuously. The examination was carried out before CAG in the morning and patients were instructed to avoid alcohol-, nicotine-, caffeine-containing products, and vasodilator drugs at least 8 h before the test. After the position of the brachial artery was located by ultrasound, the ultrasound probe was fixed and then a blood pressure cuff was placed around the upper arm distal to the brachial artery segment that was explored. The probe was angulated at 90° for optimal morphologic B-mode imaging and < 60° for optimal velocity acquisition. Brachial artery diameter and flow velocity were recorded using a UNEXEF38G vascular endothelial function detector (UNEX, Japan) before cuff inflation, at deflation, and after deflation at 1 min intervals for 5 min, and the difference between these measures represented brachial artery flow-mediated vasodilatation (FMD) value.

### Statistical analysis

IBM SPSS Statistics software (version 26.0, Chicago, USA) and R statistical analysis software (version 4.1.2, Vienna, Austria) were used for statistical analysis. Continuous variables were shown as the mean ± standard deviation for normal distribution and median with interquartile range (P25, P75) for non-normal distribution, which were compared by the Mann–Whitney U test, respectively. Categorical variables were presented as frequency (percentage) using the Chi-Square test. Clinical characteristics of included patients were compared between derivation set and validation set by univariate analysis. The least absolute shrinkage and selection operator (Lasso) regression analysis method was performed to avoid collinearity and identify key potential predictors, meanwhile, indicators with statistically significant differences were considered for the establishment of clinical prediction model. Multivariate logistic regression analysis was used to construct a clinical prediction model with identified predictors for clinical practice. Bootstrap validation was used to test performance and eventually we obtained the actual model (seed = 120, nfolds = 3). And the Hosmer–Lemeshow test was carried out to evaluate the goodness-fit of the constructed model, with lower *χ*^*2*^ and higher *P* values indicating better calibration. The area under curve (AUC) of receiver operating characteristic (ROC), calibration curve, decision curve analysis (DCA), and clinical impact curve (CIC) were plotted and utilized with validation set to comprehensively evaluate the predictive accuracy and clinical value of the model. Values for AUC range from 0.5 to 1 and the closer to unity, the more accurate a model. A difference at *P* < 0.05 was considered statistically significant.

## Results

### Selection process

**Fig. 1:**
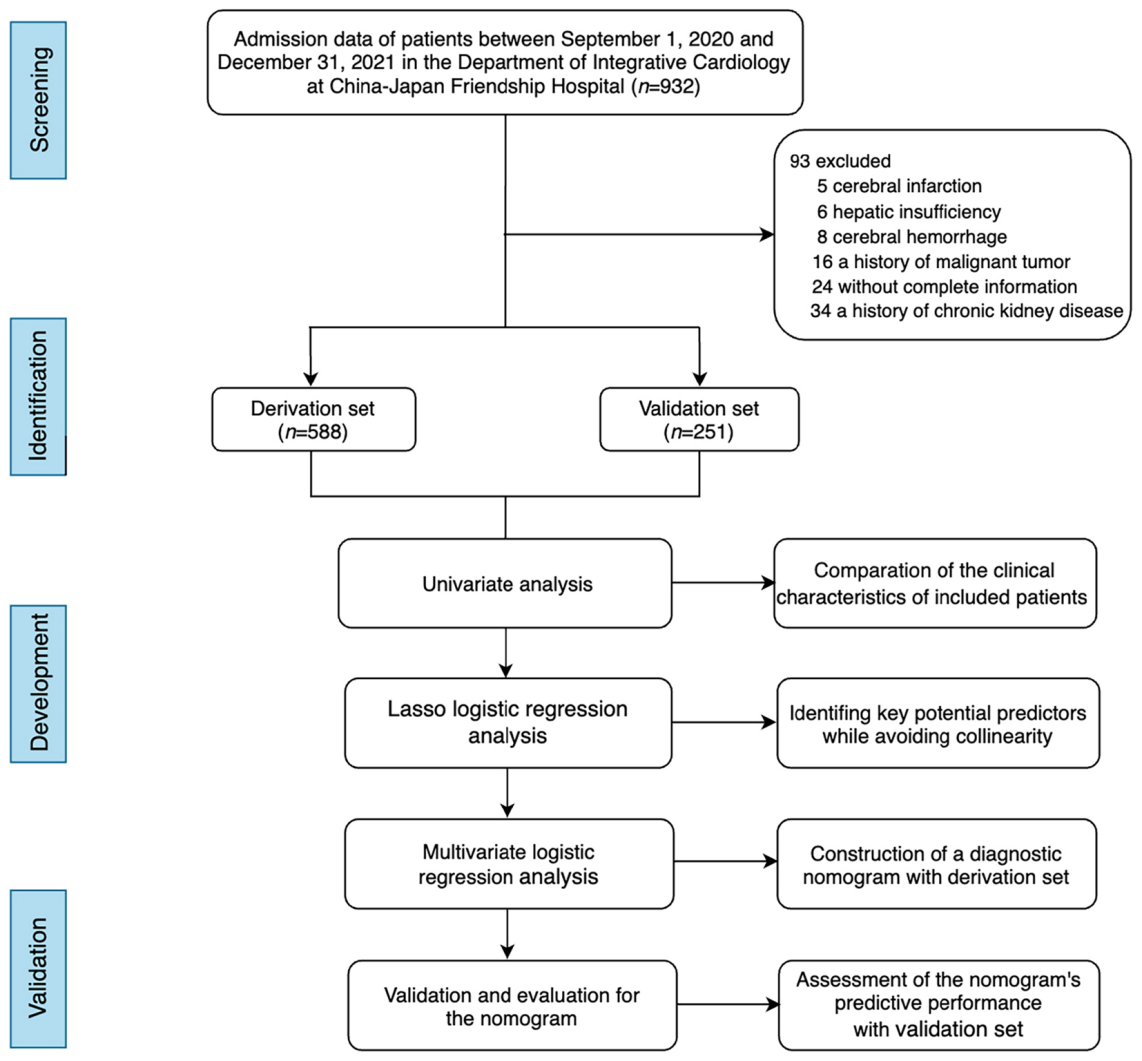
Flowchart of the detailed selection process

Admission data of 932 consecutive patients between September 1, 2020 and December 31, 2021 were retrospectively assessed in the Department of Integrative Cardiology at China-Japan Friendship Hospital. Of these, 93 were excluded according to the exclusion criteria: 16 with a history of malignant tumor, 6 with hepatic insufficiency, 34 with a history of chronic kidney disease, 8 with cerebral hemorrhage, 5 with cerebral infarction, and 24 without complete information. The rest of the patients were randomly classified into the derivation (*n* = 588) and validation sets (*n* = 251) at a 7:3 ratio and then assigned to the CHD group and non-CHD group following their CAG findings. The details of the selection process are shown in Fig. 1.

### Clinical characteristics of patients in derivation and validation sets

The baseline demographic and clinicopathologic features of patients in derivation and validation sets were listed in Table 1. Overall, there was no significant difference in gender distribution, age, BMI, proportions of smoking history, diabetes, hypertension, heart failure (NYHA I-II) and atrial fibrillation, peripheral blood parameters, Scr, Hs-CRP, blood lipid, HCY, HbA1c, NT-proBNP, FMD, as well as arterial stiffness indices and echocardiographic parameters between the two sets (*P* < 0.05). Besides, the frequency of ACEI/ARB was significantly higher than those in the validation set (*P* = 0.023).

**Table 1:** Basic characteristics of patients in the derivation set and validation set

Indicators	Derivation set (*n* = 588)	Validation set (*n* = 251)	Statistics	*P* values
Demographics				
Male, N (%)	278 (47.28)	112 (44.62)	0.499^a^	0.480
Age, years	60 (56, 67)	62 (56, 69)	-1.552^b^	0.121
BMI, kg/m^2^	25.71 (24.06, 27.84)	25.81 (24.06, 27.85)	0.016^b^	0.987
Past medical history				
Smoking, N (%)	159 (27.04)	76 (30.28)	0.915^a^	0.339
Diabetes, N (%)	250 (42.52)	107 (42.63)	0.001^a^	0.976
Hypertension, N (%)	357 (60.71)	136 (54.18)	3.096^a^	0.078
Heart failure NYHA I-II, N (%)	25 (4.25)	15 (5.98)	1.152^a^	0.283
Atrial fibrillation, N (%)	22 (3.74)	8 (3.19)	0.157^a^	0.692
Prior medication use				
Anti-platelet, N (%)	487 (82.82)	199 (79.28)	1.479^a^	0.224
Statins, N (%)	471 (80.10)	187 (74.50)	3.261^a^	0.071
ACEI/ARB, N (%)	329 (55.95)	119 (47.41)	5.158^a^	0.023
Beta-blockers, N (%)	457 (77.72)	206 (82.07)	2.009^a^	0.156
CCB, N (%)	309 (52.55)	121 (48.21)	1.329^a^	0.249
Nitrates, N (%)	265 (45.07)	98 (39.04)	2.601^a^	0.107
Laboratory values				
ALT, IU/L	20 (15, 28)	19 (14, 28)	1.086^b^	0.278
AST, IU/L	20 (15, 28)	19 (16, 23)	1.801^b^	0.072
Scr, *μ*mol/L	69 (58.3, 79.75)	67.4 (57.45, 79.65)	0.969^b^	0.333
Hs-CRP, mg/L	1.41 (0.64, 3.13)	1.55 (0.77, 3.12)	− 1.634^b^	0.102
TC, mmol/L	4.02 (3.26, 4.72)	3.95 (3.24, 4.71)	0.674^b^	0.500
TG, mmol/L	1.35 (1.04, 1.82)	1.30 (0.99, 1.84)	0.724^b^	0.469
LDL-C, mmol/L	2.4 (1.91, 2.97)	2.42 (1.93, 2.95)	0.240^b^	0.810
HDL-C, mmol/L	1.1 (0.96, 1.3)	1.13 (0.93, 1.31)	-0.004^b^	0.997
HCY, *μ*mol/L	13.37 (11.22, 15.84)	14.18 (10.77, 17.03)	− 1.385^b^	0.166
HbA1c, %	6.1 (5.6, 7)	6 (5.6, 6.8)	0.412^b^	0.681
NT-proBNP, pg/mL	80 (42, 162)	93.5 (54, 182.5)	− 1.841^b^	0.066
Arterial stiffness indices				
baPWV, m/s	17.24 (15.24, 19.80)	18.11 (15.69, 19.25)	− 1.878^b^	0.060
ABI	1.13 (1.05, 1.22)	1.12 (1.04, 1.22)	0.264^b^	0.792
Vascular endothelial function test				
FMD, %	7.7 (6.9, 9)	7.55 (6.8, 8.9)	0.861^b^	0.390
Echocardiographic values				
LAD, mm	37 (34, 40)	37 (34, 40)	− 0.870^b^	0.384
LVEF, %	68 (63.5, 71)	68 (64, 71)	− 0.810^b^	0.418
LVMI, g/m^2^	87.8 (75.73, 101.87)	88.28 (74.27, 103.71)	− 0.246^b^	0.806

Data were expressed as means ± standard deviations or as medians with interquartile ranges or as frequencies and percentages

*BMI* body mass index, *ACEI* angiotensin converting enzyme inhibitor, *ARB* angiotensin receptor blocker, *CCB* calcium channel blockers, *ALT* alanine aminotransferase, *AST* aspartate aminotransferase, *Scr* serum creatinine, *Hs-CRP* hypersensitive C-reactive protein, *TC* total cholesterol, *TG* triglyceride, *LDL-C* low-density lipoprotein cholesterol, *HDL-C* high-density lipoprotein cholesterol, *HCY* homocysteine, *HbA1c* hemoglobin A1c, *NT-proBNP* N-terminal pro-B-type natriuretic peptide, *baPWV* brachial-ankle pulse wave velocity, *ABI* ankle-brachial index, *FMD* brachial artery flow-mediated vasodilatation, *LAD* left atrium diameter, *LVEF* left ventricular ejection fraction, *LVMI* left ventricular mass index. ^a^x^2^ value, ^b^Z value

### Clinical characteristics of patients in the derivation set

Patients were classified into the CHD (N = 433) and non-CHD (N = 155) groups according to the CAG results. The baseline characteristics of patients in the two groups were compared by univariate analysis, as listed in Table 2. A total of eight indicators were identified as risk factors for the development of CHD in middle-aged and elderly people. Compared with patients in the non-CHD group, patients in the CHD group showed a higher proportion of diabetes history, as well as higher age, HbA1c, baPWV, and lower TC, LDL-C, ABI, and FMD levels (*P* < 0.001). Similar results were obtained in the validation set. (Additional file 1: Table S1).

**Table 2:** Basic characteristics of patients in the derivation set

Indicators	CHD (*n* = 433)	Non-CHD (*n* = 155)	Statistics	*P* values
Demographics				
Male, N (%)	203 (46.88)	75 (48.39)	0.104^a^	0.747
Age, years	64 (58, 69)	60 (55, 67)	3.533^b^	< 0.001
BMI, kg/m^2^	25.67 (24.03, 27.55)	26.04 (24.09, 29.07)	− 1.938^b^	0.053
Past medical history				
Smoking, N (%)	122 (28.18)	37 (23.87)	1.072^a^	0.300
Diabetes, N (%)	203 (46.88)	47 (30.32)	12.807^a^	< 0.001
Hypertension, N (%)	261 (60.28)	96 (61.94)	0.132^a^	0.717
Heart failure NYHA I-II, N (%)	18 (4.16)	7 (4.52)	0.036^a^	0.849
Atrial fibrillation, N (%)	15 (3.46)	7 (4.52)	0.351^a^	0.554
Prior medication use				
Anti-platelet, N (%)	353 (81.52)	134 (86.45)	1.948^a^	0.163
Statins, N (%)	343 (79.21)	128 (82.58)	0.811^a^	0.368
ACEI/ARB, N (%)	246 (56.81)	83 (53.55)	0.494^a^	0.482
Beta-blockers, N (%)	339 (78.29)	118 (76.13)	0.308^a^	0.579
CCB, N (%)	237 (54.73)	72 (46.45)	3.140^a^	0.076
Nitrates, N (%)	201 (46.42)	64 (41.29)	1.213^a^	0.271
Laboratory values				
ALT, IU/L	20 (15, 28)	20 (14, 28.5)	− 0.332^b^	0.740
AST, IU/L	19 (16, 24)	20 (15.5, 23)	− 0.576^b^	0.565
Scr, *μ*mol/L	69.3 (58.3, 81.05)	67.3 (59.3, 78.5)	0.969^b^	0.333
Hs-CRP, mg/L	1.32 (0.65, 3.33)	1.48 (0.62, 2.57)	0.762^b^	0.446
TC, mmol/L	3.87 (3.22, 4.63)	4.33 (3.48, 4.88)	− 3.192^b^	0.001
TG, mmol/L	1.34 (1.06, 1.8)	1.43 (1.03, 1.88)	− 0.103^b^	0. 918
LDL-C, mmol/L	2.34 (1.88, 2.93)	2.53 (2.08, 3.12)	− 3.082^b^	0.002
HDL-C, mmol/L	1.09 (0.96, 1.27)	1.15 (1, 1.34)	− 1.748^b^	0.080
HCY, *μ*mol/L	13.49 (11.26, 16.2)	13.27 (11.24, 15.03)	1.287^b^	0.198
HbA1c, %	6.2 (5.7, 7.2)	5.8 (5.5, 6.2)	5.053^b^	< 0.001
NT-proBNP, pg/mL	91.5 (47.5, 176.5)	78 (40, 134)	1.844^b^	0.065
Arterial stiffness indices				
baPWV, m/s	17.81 (15.69, 20.02)	16.38 (13.89, 19.05)	4.641^b^	< 0.001
ABI	1.12 (1.02, 1.21)	1.15 (1.08, 1.22)	− 2.445^b^	0.014
Vascular endothelial function test				
FMD, %	7.6 (6.8, 9)	8 (7.2, 9.1)	− 2.181^b^	0.029
Echocardiographic values				
LAD, mm	37 (34, 40)	36 (34, 40)	1.085^b^	0.278
LVEF, %	68 (63, 71)	68 (64, 72)	-1.220^b^	0.223
LVMI, g/m^2^	88.25 (76.3, 102.26)	85.18 (71.75, 99.79)	1.386^b^	0.166

Data were expressed as means ± standard deviations or as medians with interquartile ranges or as frequencies and percentages

*CHD* coronary atherosclerotic heart disease, *BMI* body mass index, *ACEI* angiotensin converting enzyme inhibitor, *ARB* angiotensin receptor blocker, *CCB* calcium channel blockers, *ALT* alanine aminotransferase, *AST* aspartate aminotransferase, *Scr* serum creatinine, *Hs-CRP* hypersensitive C-reactive protein, *TC* total cholesterol, *TG* triglyceride, *LDL-C* low-density lipoprotein cholesterol, HDL*-C* high-density lipoprotein cholesterol, *HCY* homocysteine, *HbA1c* hemoglobin A1c; *NT-proBNP* N-terminal pro-B-type natriuretic peptide, *baPWV* brachial-ankle pulse wave velocity, *ABI* ankle-brachial index, *FMD* brachial artery flow-mediated vasodilatation, *LAD* eft atrium diameter, *LVEF* left ventricular ejection fraction, *LVMI* left ventricular mass index

^a^*χ*^*2*^ value

^b^*Z* value

### Lasso regression analysis to identify key potential predictors

**Fig. 2:**
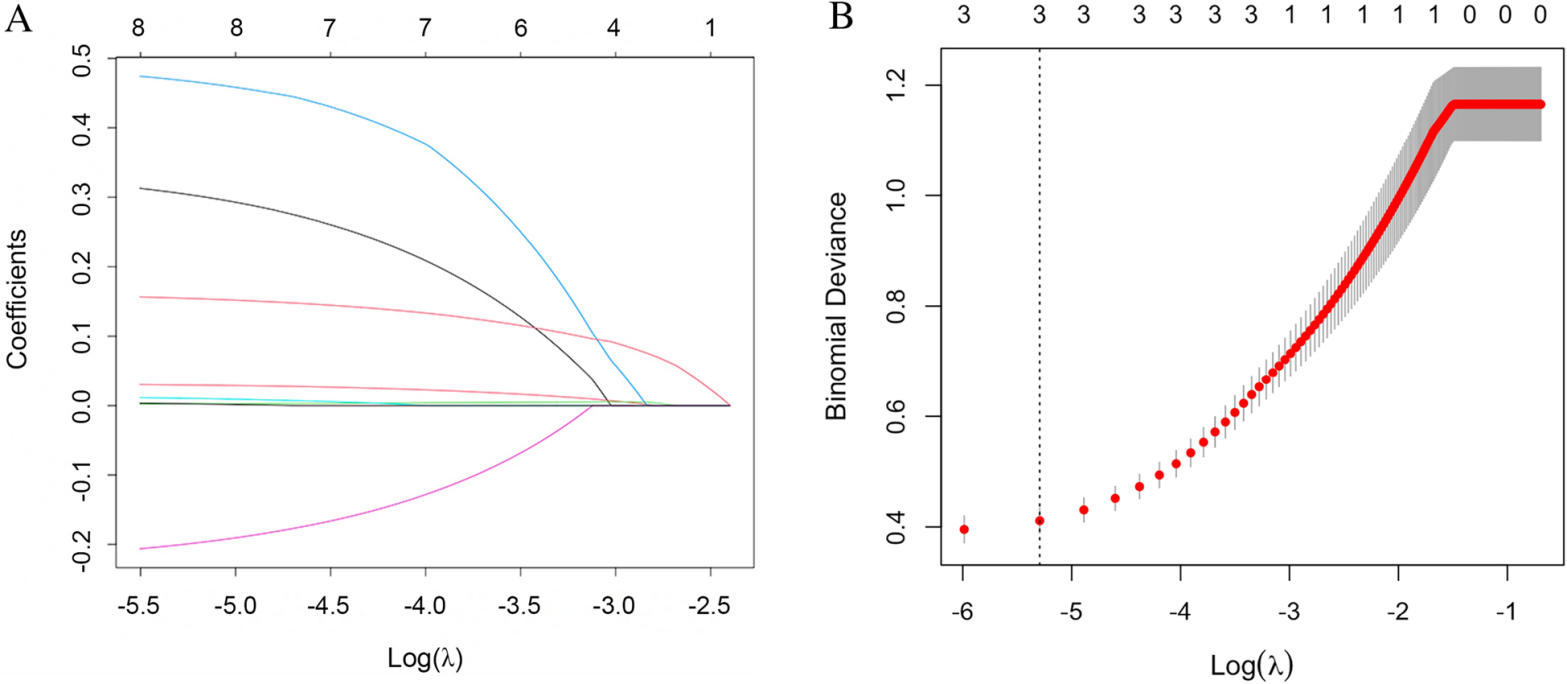
The process of selecting key potential predictors by Lasso regression analysis. **A** Coefficients profile of selected predictors using Lasso regression analysis; **B** Using all the sample and candidate predictors, we employ Lasso to select the primitive predictors by cross-validation method

Lasso regression analysis method was performed to avoid collinearity and identify key potential predictors. On this basis, variables were again selected using a regression model, and those with *P* < 0.05 were integrated into the analysis and considered for the establishment of clinical prediction model. Furthermore, we then used Lasso regression simulations to assess eight candidate variables, with non-zero coefficients taking a penalty paramete (*λ*)., as shown in Fig. 2. Meanwhile, indicators with statistically significant differences were considered for the establishment of clinical prediction model. Finally, four key potential predictors were identified combined with the results of univariate analysis, including age, HbA1c, ABI, and FMD (*λ* = 0.005). These four key potential predictors would be further incorporated into the development of the model in the next step.

### Construction of the diagnostic nomogram by multivariate logistic regression analysis

**Fig. 3:**
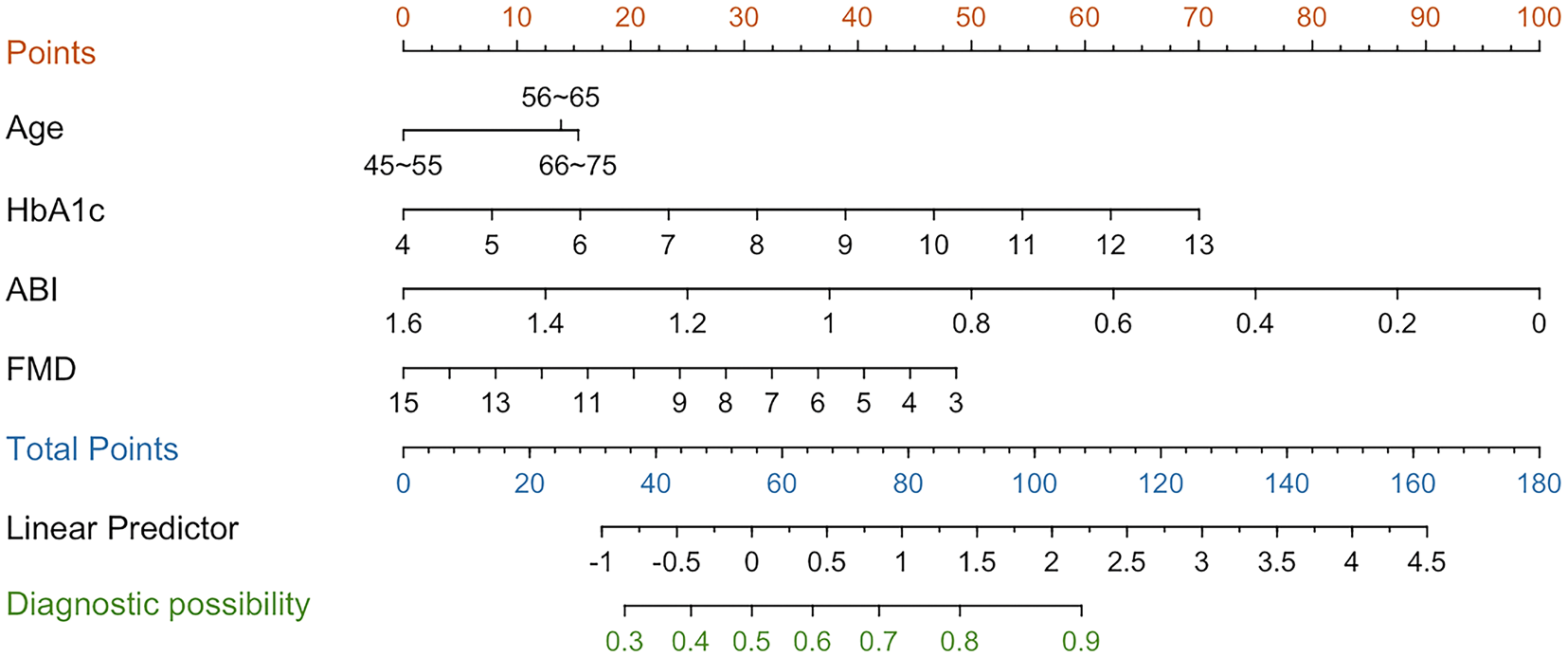
Nomogram predicting CHD in middle-aged and elderly people

By the previous univariate analysis, the presence of diabetes history, age, HbA1c, baPWV, and lower TC, LDL-C, ABI, and FMD levels were identified as risk factors for the development of CHD in middle-aged and elderly people. Of these parameters, age (OR 1.034, 95% CI 1.002 ~ 1.067, *P* = 0.040), HbA1c (OR 1.380, 95% CI 1.078 ~ 1.768, *P* = 0.011), ABI (OR 0.078, 95% CI 0.012 ~ 0.522, *P* = 0.009), and FMD (OR 0.848, 95% CI 0.726 ~ 0.990, *P* = 0.037) levels were identified as independent predictors for CHD development on multivariate logistic regression analysis, as shown in Table 3. Based on this result, a new predictive equation was established: Risk score = 1.451 + 0.033*Age + 0.322*HbA1c-2.548*ABI-0.165*FMD. The nomogram was incorporated with the above independent four predictors, as shown in Fig. 3. The weights of predictors are represented by the length of the line segment, which was positively correlated with the degree to which the predictors affected clinical outcomes. The diagnostic possibility corresponds to the total points by adding the scores for each predictor, indicating the probability of CHD.

**Table 3:** Multivariate logistic regression analysis of independent risk factors

Indicators	*β*	SE	Wald	*P* values	OR	95% *CI*
Age	0.033	0.016	4.219	0.040	1.034	[1.002, 1.067]
HbA1c	0.322	0.126	6.520	0.011	1.380	[1.078, 1.768]
ABI	− 2.548	0.969	6.922	0.009	0.078	[0.012, 0.522]
FMD	− 0.165	0.079	4.364	0.037	0.848	[0.726, 0.990]
Constant	1.451	1.874	0.599	0.439	4.267	

*β* regression coefficient, *SE* standard error, Wald, *χ*^*2*^ value, *OR* odds ratio, *CI* confidence interval. HbA1c, hemoglobin *A1c*, ABI, ankle-brachial index, *FMD* brachial artery flow-mediated vasodilatation

**Fig. 4:**
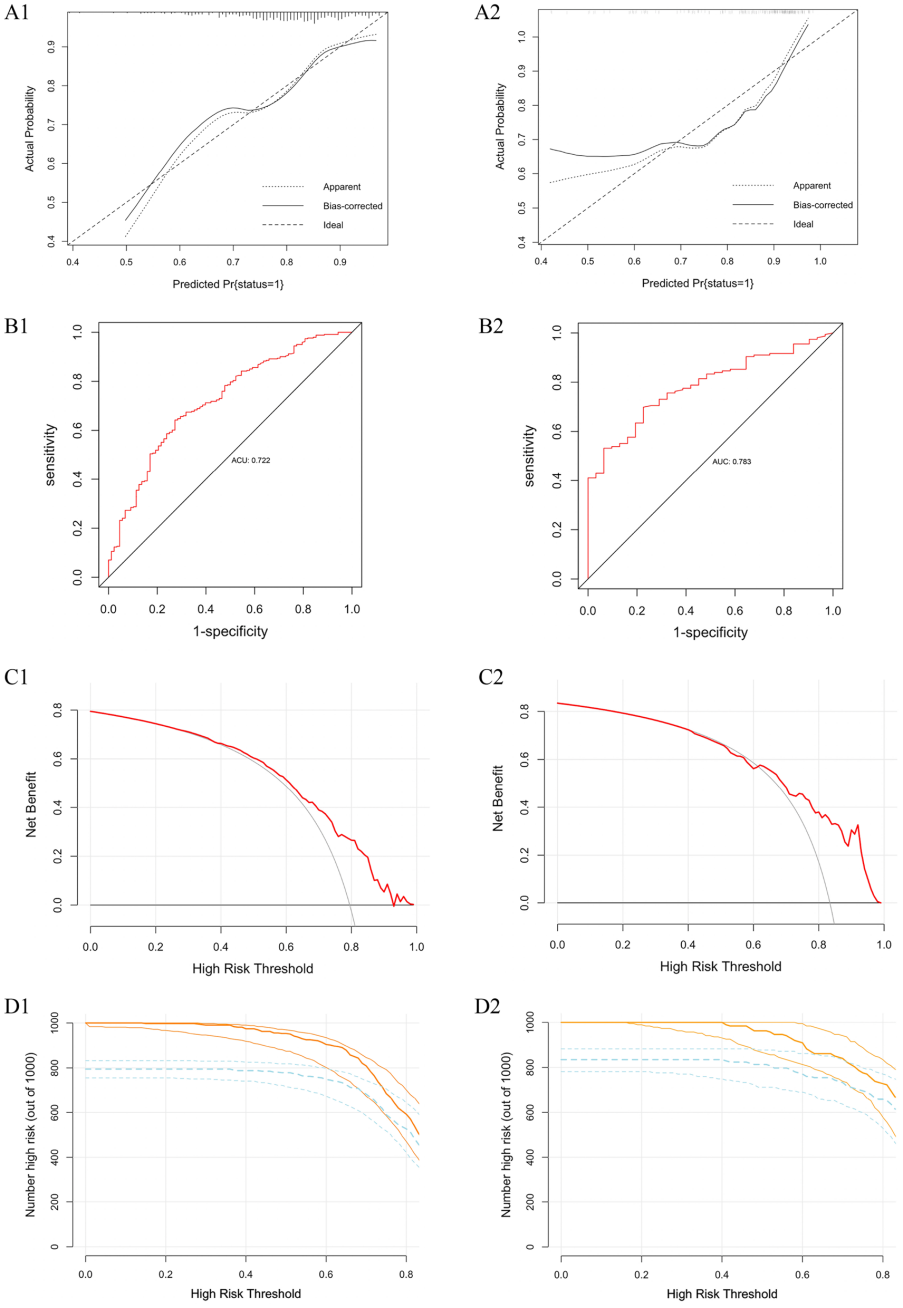
Apparent performance of the prediction model in the derivation set and validation set. **A1** Calibration curve of the multivariate prediction model in the derivation set. **B1** ROC curve of the multivariate prediction model in the derivation set. **C1** DCA of the model in the derivation set: Y-axis represents the net benefit. The red solid line represents the CHD prediction model, the thin solid line is the hypothesis that all patients get achievement of CHD and receive treatment, and the thick one is the assumption that no patients have CHD and none receive treatment. **D1** CIC of the model in the derivation set. The yellow solid line represents the number of high-risk patients and the blue dotted line is the number of high-risk patients with events in the 1000 patients.**A1**Calibration curve of the multivariate prediction model in the validation set. (B2) ROC curve of the multivariate prediction model in the validation set. **C1** DCA of the multivariate prediction model in the validation set. **C1** CIC of the multivariate prediction model in the validation set

Meanwhile, the Hosmer–Lemeshow test showed a good calibration performance of the clinical prediction model (*χ*^*2*^ = 7.865, *P* = 0.447), and the calibration curve indicated an excellent consistency of the model in the derivation set (Fig. 4A1). The ROC curve demonstrated the predictive power of the nomogram with an AUC value of 0.722 (Fig. 4B1), indicating that the nomogram had a high predictive value. In addition, DCA and CIC analysis was ultilized to assess the clinical utility of the prediction model (Fig. 4C1, D1), suggesting that the model had a good overall net benefit and clinical impact within most reasonable threshold probability.

### Performance of the clinical prediction model

Validation and evaluation of performance of the clinical prediction model was conducted using Bootstrap method. Admission data of 251 eligible patients was collected and analyzed for the internal validation of the prediction model. The results of calibration plot, AUC value, DCA analysis, and CIC analysis stemmed from the validation set were similar to those from the derivation set. In the validation set, the goodness-of-fit *χ*^*2*^ of CHD in middle-aged and elderly people was 11.132 (*P* = 0.194), which indicated no evidence of poor fit between observation and prediction (Fig. 4A2). Besides, the ROC curve revealed an AUC value of 0.783 (Fig. 4B2). Moreover, the nomogram demonstrated a high net benefit in predicting the CHD probability among middle-aged and elderly people by DCA and CIC analysis (Fig. 4C2, D2). Overall, these results showed that the novel nomogram had a good predictive power and clinical utility for the prediction of CHD probability in middle-aged and elderly people.

## Discussions

Recently, with the growing morbidity and mortality related to CHD in middle-aged and elderly people, the early diagnosis and treatment of CHD have received extensive attention worldwide [3] Therefore, a new prediction model for CHD patients could be the key to early screening and diagnosis and thus improve their prognosis. In this study, we attempted to construct and verify a diagnostic model based on easily available parameters such as data on demographics, complications, clinical and laboratory indicators at baseline. A total of 932 consecutive patients with suspected CHD were retrospectively evaluated, and 839 eligible patients were enrolled in the analysis. Eight indicators were recognized as risk factors for the progression of CHD in the derivation set, of these predictors, age, HbA1c, ABI, and FMD defined to be significantly related to CHD in middle-aged and elderly people by Lasso regression analysis. Moreover, the performance of the clinical prediction model was validated in the validation set, showing high net benefit, good ability, and great clinical utility of the model according to the results of calibration plot, AUC value, DCA analysis, and CIC analysis.

This study showed that predictors such as age, HbA1c, ABI, and FMD were combined as independent risk factors in the prediction model. The main reason is that CHD is a disease caused by a variety of risk factors, which is thought to be mainly associated with age, glucose metabolism, and vascular health [1] Additionally, the result is similar to some previous reports on the risk factors of CHD [17–19] However, fewer concerned ABI and FMD, which are associated with peripheral artery disease (PAD), in previous models focusing on CHD. These indicators are easy to acquire in the electronic medical systems of inpatients, strengthening the ease of use and comprehensiveness of the prediction model.

Consistent with previous findings, our results demonstrated that the older the patients, the higher was the risk score in the nomogram. In detail, prevalent cases of CHD began to account for a large proportion of epidemic cases of cardiovascular disease in patients over 40 years old, and the prevalence rose steeply with elder age categories [4] According to the report [3] there were approximately 10.88 million prevalent cases of CHD in patients aged 50 to 54 years, more than three times the number of cases in patients aged 40 to 44 years.

At present, the relationship between risk factors associated with diabetes and PAD, and the occurrence and development of CHD in middle-aged and elderly people are gradually obtaining more attention [1, 20, 21] In the derivation set, patients in the CHD group had a higher HbA1c level than those in the non-CHD group, indicating that HbA1c is positively associated with the risk of CHD. In addition, a high HbA1c level was shown to be an important risk factor for glucose metabolism progression in cardiovascular disease [22, 23] Knowledge of glucose metabolism is significant on account of a well-established link between adverse cardiovascular outcomes and diabetes [24–26] Meanwhile, ordinary measurement of HbA1c in every patient with suspected CHD was recommended [1] As outlined in the 2019 ESC Guidelines [21] targeting near-normal HbA1c for glycemic control will reduce cardiovascular complications in diabetic patients, and less-rigorous HbA1c goals may be more appropriate for senile patients with severe comorbidities on a personalized basis. Besides, it has been shown that a reduction of approximately 1% in HbA1c was associated with a 15% reduction in the relative risk of non-fatal myocardial infarction [27] and proper glycemic control at an early stage is strongly related to long-term cardiovascular benefits [28].

It confirmed that the risk of death derived from cardiovascular causes is at a higher level in patients with large-vessel PAD [29] The ABI is a sensitive marker for arterial stiffness [30] and the FMD can be tested noninvasively to evaluate vascular endothelial function associated with atherosclerosis [31] In the derivation set, patients in the CHD group had lower ABI and FMD levels than those in the non-CHD group, indicating that the ABI and FMD levels were negatively correlated with the risk of CHD. Meanwhile, this study showed that the lower the levels, the higher was the risk score in the nomogram. Indeed, impaired ABI and FMD have been reported as early biomarkers of the development of atherosclerotic disease in previous studies, and higher values generally predict better coronary vascular outcomes [32, 33] A previous report found that patients with an FMD ≥ 10% were less strongly associated with fewer cardiovascular risk factors than those with an FMD < 10% [31] Moreover, ABI may be defined as a risk modifier in the assessment of cardiovascular risk [34] According to the American Heart Association, [35] ABI is an independent predictor of the cardiovascular event risk, even in the absence of PAD symptoms. The degree of the increased risk related to a low ABI is approximately two to three times greater in patients with diagnosed cardiovascular disease than in healthy individuals, and a decline in ABI of > 0.15 over time is related to a twofold increase in mortality [30, 35]

Among the present studies, cardiovascular risk prediction models involving traditional risk factors such as sex, age, smoking history, hypertension, diabetes mellitus, and hypercholesterolemia have been utilized to evaluate risk of future cardiovascular events [36] These prediction models, however, have limited comprehensiveness and accuracy [37] resulting in the assessment of other risk predictors such as electrocardiogram [38] or parameters of obesity such as waist circumference, [17] used in combination with other traditional risk factors or alone. More accurate recognization of high-risk individuals could facilitate the development of appropriate targeted aggressive risk reduction therapies, but more proper assessments for this strategy are still required in the future.

Furthermore, both the Hosmer–Lemeshow test and the calibration curve showed a good consistency between the actual and predicted risk of CHD, which ensures the reliability and repeatability of the CHD prediction model. Meanwhile, the discrimination of the model was assessed by the AUC value. The AUC value was 0.722 in the derivation set for the model to predict functional outcome and 0.783 in the validation set, showing that the clinical prediction model had a good predictive ability [39] Besides, the results of DCA and CIC analysis illustrated that the clinical prediction model had remarkable predictive power. DCA analysis was utilized to assess the clinical utility of the prediction models, in which the net benefit is defined as the difference between the expected benefit and the expected harm of each prediction model [40] The plots indicated that the clinical prediction model showed a greater net benefit with a wider range of threshold probabilities for predicting CHD in the two sets. The DCA for the derivation set indicated that the net benefit was maximized with threshold probabilities of 0% to 40% by the “predict all” method. Moreover, the CIC was further mapped on the basis of the DCA to assess the clinical impact, presenting the approximate number of patients with predicted CHD and the number of those who were in actual situation of illness at each risk threshold. When the risk threshold is greater than 60%, the estimated value is closer to the true number. Meanwhile, a similar trend was seen in the validation set.

The study is the first to construct and validate a clinical prediction model of CHD and investigate the value of easily accessible clinical and laboratory predictors to predict the development of CHD in middle-aged and elderly people. In addition, the nomogram we developed is beneficial to the early diagnosis of CHD, especially those who are not suitable for CAG or constrained by patients' characteristics such as those with severe comorbidities or the absence of medical conditions. Its purpose is to help clinicians make appropriate clinical treatment decisions based on individual conditions.

## Limitations

Nevertheless, certain limitations of the study need to be mentioned. Firstly, this study is a retrospective single-center study with a single population source leads to a certain degree of selection bias. The study only included patients with suspected CHD, which may restrict the generalizability of the findings to patients with confirmed CHD or those without suspected CHD. Secondly, this study adopted a single time-node data modeling, which could not avoid the impact of dynamic changes in the physiological and pathological states of patients over time on the model. Subsequently, the design could be further improved, and multi-time-node data could be collected and analyzed to improve and update the model. Thirdly, the clinical performance of the constructed model was only evaluated by internal validation, therefore, the clinical value in external application needs to be further verified. The study did not assess the impact of the prediction model on patient outcomes, such as mortality or morbidity, which could help providing a more comprehensive evaluation of the model's clinical utility. We look forward to more large-scale, multi-center and prospective studies with rigorous and standardized design to verify and improve the results of this study.

## Conclusion

This study established and validated a clinical prediction model for several clinical predictors related to the diagnosis probability of CHD in middle-aged and elderly people, including age, HbA1c, ABI, and FMD. The performance of the model was determined to be of high net benefit, strong ability, and great clinical utility in the validation set. The results could potentially contribute to the early diagnosis and treatment of CHD in middle-aged and elderly people, which may ultimately improve the prognosis of patients with CHD.

### Supplementary Information

**Additional file 1: Table S1.** Basic characteristics of patients in the validation set.

## Data Availability

All data generated or analyzed during this study are included in this published article and its additional information files.
