# Supplementary material for: Development and validation of a clinical prediction model for detecting coronary heart disease in middle-aged and elderly people: a diagnostic study
Source: Eur J Med Res. 2023 Sep 25;28:375. doi: 10.1186/s40001-023-01233-0 (PMC10521501; doi:10.1186/s40001-023-01233-0)
Supplement: Supplementary file 1 — Additional file 1: Table S1. Basic characteristics of patients in the validation set. [file 40001_2023_1233_MOESM1_ESM.pdf]

Additional file materials

Table S1 Basic characteristics of patients in the validation set

Indicators	CHD (*n*=185)	Non-CHD (*n*=66)	Statistics	*P* values
**Demographics**				
Male, N (%)	84 (45.41)	28 (42.42)	0.175[a]	0.676
Age, years	66 (56.5, 75)	64 (56, 69)	2.441[b]	0.015
BMI, kg/m^2	25.77±2.34	26.48±2.89	2.846[b]	0.093
**Past medical history**				
Smoking, N (%)	57 (30.81)	19 (28.79)	0.094[a]	0.759
Diabetes, N (%)	80 (43.24)	27 (40.91)	0.108[a]	0.742
Hypertension, N (%)	91 (49.19)	35 (53.03)	0.287[a]	0.592
Heart failure NYHA I-II, N (%)	12 (6.49)	3 (4.55)	0.326[a]	0.568
Atrial fibrillation, N (%)	7 (3.78)	1 (1.52)	0.811[a]	0.368
**Prior medication use**				
Anti-platelet, N (%)	149 (80.54)	50 (75.76)	0.678[a]	0.410
Statins, N (%)	140 (75.68)	47 (71.21)	0.510[a]	0.475
ACEI/ARB, N (%)	87 (47.03)	32 (48.48)	0.041[a]	0.839
Beta-blockers, N (%)	152 (82.16)	54 (81.82)	0.004[a]	0.950
CCB, N (%)	85 (45.95)	36 (54.55)	1.441[a]	0.230
Nitrates, N (%)	75 (40.54)	23 (34.85)	0.662[a]	0.416
**Laboratory values**				
ALT, IU/L	17 (13, 24)	21 (17, 30.5)	−0.816[b]	0.414
AST, IU/L	18 (15, 22)	17 (14.5, 21.5)	0.981[b]	0.327
Scr, μmol/L	62.4 (55.55, 73.35)	65.9 (54.25, 76.35)	1.052[b]	0.293
Hs-CRP, mg/L	1.47 (0.76, 2.72)	1.54 (0.62, 2.573)	0.866[b]	0.387
TC, mmol/L	3.97±1.09	4.12±0.95	1.407[b]	0.237
TG, mmol/L	1.26 (0.98, 1.8)	1.36 (1.06, 1.95)	−2.103[b]	0.035
LDL-C, mmol/L	2.43±0.8	2.56±0.69	2.110[b]	0.148
HDL-C, mmol/L	1.17 (0.96, 1.38)	1.03 (0.91, 1.29)	−0.409[b]	0.683
HCY, μmol/L	12.7 (9.81, 15.84)	13.58 (10.94, 14.99)	0.507[b]	0.612
HbA1c, %	6.2 (5.7, 7.55)	5.7 (5.6, 6.25)	2.418[b]	0.016
NT-proBNP, pg/mL	80 (54, 90)	70 (36.5, 94.5)	1.659[b]	0.097
**Arterial stiffness indices**				
baPWV, m/s	19.85 (16.54, 23)	17.5 (15.89, 20.07)	2.345[b]	0.019
ABI	1.12 (1.04, 1.22)	1.15 (1.09, 1.29)	−2.621[b]	0.009
**Vascular endothelial function test**				
FMD, %	7.2 (6.7, 8.4)	9.1 (8.35, 10)	−5.092[b]	<0.001
**Echocardiographic values**				
LAD, mm	37.7±5.18	37.12±4.02	2.748[b]	0.099
LVEF, %	69 (65, 72)	69 (66.5, 71.5)	−1.926[b]	0.054
LVMI, g/m^2	90.9±23.61	88.78±21.44	1.691[b]	0.195

Data were expressed as means±standard deviations or as medians with interquartile ranges or as frequencies and percentages

CHD, coronary atherosclerotic heart disease; *BMI*, body mass index; *ACEI*, angiotensin converting enzyme inhibitor; *ARB*, angiotensin receptor blocker; *CCB*, calcium channel blockers; *ALT*, alanine aminotransferase; *AST*, aspartate aminotransferase; *Scr*, serum creatinine; *Hs-CRP*, hypersensitive C-reactive protein; *TC*, total cholesterol; *TG*, triglyceride; *LDL-C*, low-density lipoprotein cholesterol; *HDL-C*, high-density lipoprotein cholesterol; *HCY*, homocysteine; *HbA1c*, hemoglobin A1c; *NT-proBNP*, N-terminal pro-B-type natriuretic peptide; *baPWV*, brachial-ankle pulse wave velocity; *ABI*, ankle-brachial index; *FMD*, brachial artery flow-mediated vasodilatation; *LAD*, left atrium diameter; *LVEF*, left ventricular ejection fraction; *LVMI*, left ventricular mass index; [a]χ^2 value; [b]*Z* value
